# The Mechanism of Room-Temperature Ionic-Liquid-Based Electrochemical CO_2_ Reduction: A Review

**DOI:** 10.3390/molecules22040536

**Published:** 2017-03-28

**Authors:** Hyung-Kyu Lim, Hyungjun Kim

**Affiliations:** Graduate School of EEWS, Korea Advanced Institute of Science and Technology, 291 Daehak-ro, Yuseong-gu, Daejeon 34141, Korea

**Keywords:** room-temperature ionic-liquid, carbon dioxide, electrocatalytic system, faradic efficiency, product selectivity

## Abstract

Electrochemical CO_2_ conversion technology is becoming indispensable in the development of a sustainable carbon-based economy. While various types of electrocatalytic systems have been designed, those based on room-temperature ionic liquids (RTILs) have attracted considerable attention because of their high efficiencies and selectivities. Furthermore, it should be possible to develop more advanced electrocatalytic systems for commercial use because target-specific characteristics can be fine-tuned using various combinations of RTIL ions. To achieve this goal, we require a systematic understanding of the role of the RTIL components in electrocatalytic systems, however, their role has not yet been clarified by experiment or theory. Thus, the purpose of this short review is to summarize recent experimental and theoretical mechanistic studies to provide insight into and to develop guidelines for the successful development of new CO_2_ conversion systems. The results discussed here can be summarized as follows. Complex physical and chemical interactions between the RTIL components and the reaction intermediates, in particular at the electrode surface, are critical for determining the activity and selectivity of the electrocatalytic system, although no single factor dominates. Therefore, more fundamental research is required to understand the physical, chemical, and thermodynamic characteristics of complex RTIL-based electrocatalytic systems.

## 1. Introduction

CO_2_ conversion technology is a valuable method that converts CO_2_, a greenhouse gas, into useful carbon resources such as fuels and fine chemicals [1,2,3]. This technology is not only important scientifically but is also vital for a sustainable future. The various ongoing investigations can be categorized as biochemical [4,5,6,7,8], thermochemical [9,10,11,12,13], photochemical [14,15,16,17], and electrochemical approaches [18,19,20,21,22,23]. Among these, the electrochemical method shows the most promise as an efficient form of CO_2_ conversion technology, because of its many advantages like high reactivity under ambient conditions and good extensibility from small- to large-scale processes [19]. Moreover, an ideal, closed-loop carbon cycle, from feedstock to energy production, is possible by directly integrating the electrochemical CO_2_ conversion system with renewable sources of electricity [24,25,26].

Generally, an electrochemical CO_2_ conversion system is composed of a cathode part where the CO_2_ molecule is directly reduced to other chemicals (e.g., CO, CH_3_OH and C_2_H_4_ etc.) on the catalyst surface (e.g., Ag, Au, Cu, and Zn etc.), and an anode part where counter oxidation reaction takes place (e.g., water oxidation in aqueous system). These two cathode and anode parts are present together in the same reaction medium or separated by a proton exchange membrane to take an advantage of independent variation and optimization for each part. Although there have been many investigations on the development of high-performance electrochemical CO_2_ conversion systems, these systems have not yet reached a commercial level regarding efficiency, product selectivity, and stability. Here, the most critical bottleneck is the high thermodynamic stability of CO_2_ gas [27]. This bottleneck means that electron injection into CO_2_, which is the first reduction step in electrochemical CO_2_ conversion, is significantly disfavored (e.g., reduction at −1.9 V vs. standard hydrogen electrode (SHE) in dimethylformamide [28]). Many types of electrocatalytic system have been proposed to resolve this problem, from traditional transition-metal-based catalysts to complex biomimetic systems [29,30,31,32]. In particular, Rosen et al. reported a highly efficient Ag-based electrocatalytic system with a reaction medium comprising 1-ethyl-3-methylimidazolium tetrafluoroborate ([Emim][BF_4_]), one of the room-temperature ionic-liquids (RTILs), and water [33]. This result was notable because the reaction medium had a significant impact on the catalytic reactivity, thereby expanding the investigative scope from solely catalyst design to the design of the reaction environment, especially that of the RTIL.

RTILs are organic salts that consist of ionic species in the liquid state, even at room temperature. As such, the properties of RTILs are different from conventional aqueous or non-aqueous solvents and are characterized by their nearly-zero vapor pressures, high electrical conductivities, high electrical and thermal stabilities, and high solubilities [34,35,36,37]. In particular, a significant advantage of RTILs is their tunability, which is made possible through the many possible combinations and chemical modifications of the cations and anions. Therefore, many researchers have tried to find novel electrocatalytic systems based on RTILs by varying the type and components of the catalyst and the reaction medium [38,39,40,41]. In contrast, for the rational design of an RTIL-based electrocatalytic system with improved performance, an understanding of the intrinsic character and the underlying mechanism of electrochemical CO_2_ reduction in RTIL-based catalytic systems is crucial. Consequently, significant research in this area has been carried out. However, the understanding of RTIL-based electrochemical CO_2_ reduction systems is still limited because the reaction system involves many types of non-neutral and complex chemical components, and the reaction occurs in a very thin electrode–solvent interfacial region, i.e., the electrical double layer region [42,43,44,45]. Thus, there is no unified experimental or theoretical design principle for RTIL-based electrochemical CO_2_ reduction systems.

This mini-review aims to summarize recent experimental and theoretical studies concerning the CO_2_ reduction mechanism in RTIL-based media and to provide an overall insight and guidelines for the design of new electrocatalytic systems. The review is divided into two parts. First, approaches focusing on the electrode–RTIL interface are summarized, and, in the second part, we have summarized studies concerning the correlation between the activity and the molecular structure of the RTIL components.

## 2. The Roles of RTILs at the Electrode–RTIL Interface

**Figure 1:**
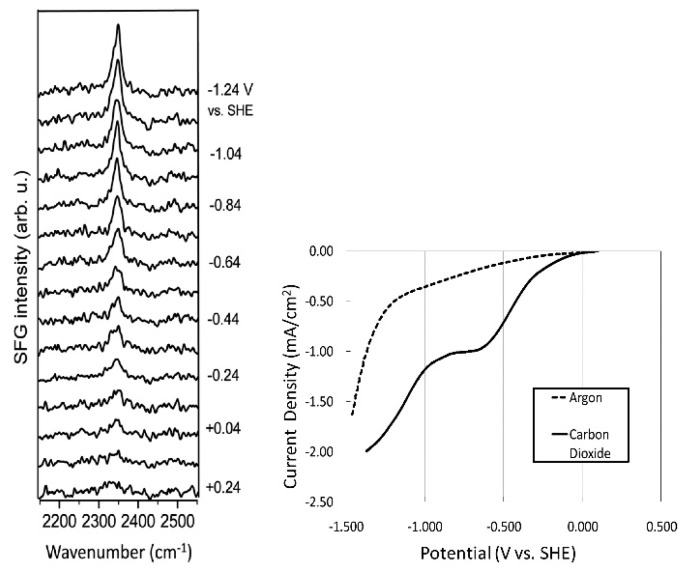
Series of SFG spectra taken during CO_2_ electrolysis in [Emim][BF_4_] containing 90 mM water (**left**); Linear sweep voltammogram of three electrode cell taken simultaneously with SFG spectra (**right**). The current in the “argon” spectrum is attributed to reduction residual water in our [Emim][BF_4_] because pure [Emim][BF_4_] has been reported to be stable between +2 and −2 V versus SHE, and the residual current varies with the water concentration. Reprinted with permission from ref. [46]. Copyright 2012 American Chemical Society.

Following the seminal work by Rosen et al. [33], they carried out various experimental characterizations to identify the underlying CO_2_ reduction mechanism [46,47,48]. In particular, vibrational spectroscopy techniques have been used to reveal the characteristics of the solid–liquid interface; furthermore, sum frequency generation (SFG), a powerful technique for the exploration of molecular structures at interfaces, has proven useful [49,50,51]. In the SFG spectrum of a system comprising a Pt electrode and [Emim][BF_4_], Rosen et al. observed CH_3_ bending (~1430 cm^−1^) and ring stretching (~1570 cm^−1^) modes [46]. This implies that [Emim] cations are preferably located at the electrode surface during electrolysis. Moreover, a series of SFG spectra measured during the electrochemical CO_2_ reduction reaction (Figure 1) show that when the cathode potential becomes more negative, a sharp peak gradually appears at 2348 cm^−1^, attributed to the formation of an [Emim]-CO_2_-[BF_4_] complex at the electrode surface. Since this peak is slightly shifted from the stretching mode of gas phase CO_2_ (2396 cm^−1^), the authors suggested that CO_2_ is not adsorbed onto the electrode surface (linear CO_2_ is not SFG-active) but the [Emim] binds to CO_2_, which results from electron transfer at the electrode surface. Braunschweig et al. also suggested the complex formation of [Emim] and CO_2_ at the interface [52].

**Figure 2:**
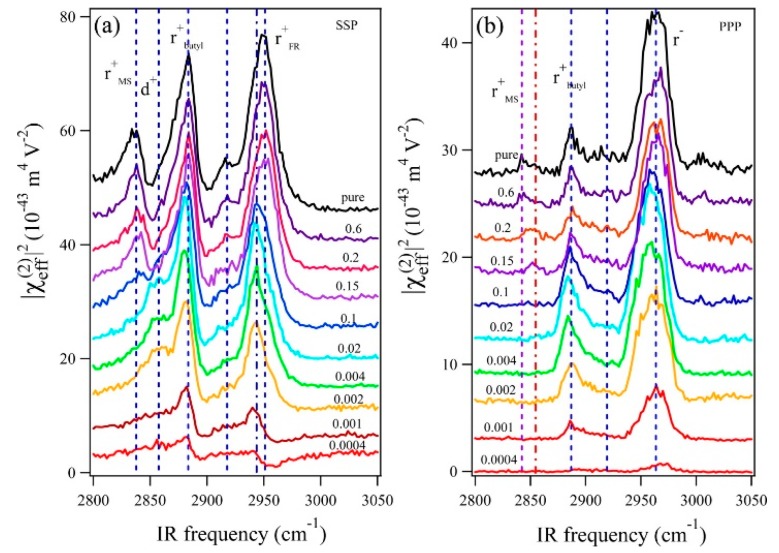
SFG spectra of [Bmim][MS] aqueous solutions with different [Bmim][MS] mole fractions (0.0004, 0.001, 0.002, 0.004, 0.02, 0.1, 0.15, 0.2, 0.6, and 1): (**a**) SSP and (**b**) PPP polarization combinations. The spectra for different [Bmim][MS] mole fractions are offset for clarity. The shape and intensity of the SSP spectra significantly change as the mole fraction of [Bmim][MS] increases. The SSP spectra show that the peak of the anion (2845 cm^−1^) appears when the mole fraction of [Bmim][MS] reaches 0.1, suggesting that anions adsorb to the surface. Reprinted with permission from ref. [53]. Copyright 2016 American Chemical Society.

Deng et al. investigated the adsorption behavior of the cations and anions of 1-butyl-3-methylimidazolium methylsulfate ([Bmim][MS]) in aqueous solution at the air–liquid surface, and the relationship between the surface structure and surface tension in terms of the RTIL concentration in solution [53]. Although this experiment was performed with an air–liquid interface instead of an electrode–solvent interface, the results provide important insights into the molecular structures and their correlation with the physical properties. Using the SFG spectra of aqueous [Bmim][MS] measured at different mole fractions (Figure 2), the authors found that the amphiphilic imidazolium cation is surface-active and behaves like a surfactant even at very low mole fractions. As the mole fraction of the RTIL increased to 0.1, the surface became saturated with [Bmim], and [MS] began to adsorb to the [Bmim] surface layer. Since the surface tension rapidly decreased at low concentrations, the authors suggested that the cations affect the surface tension to a greater extent than the anions.

**Figure 3:**
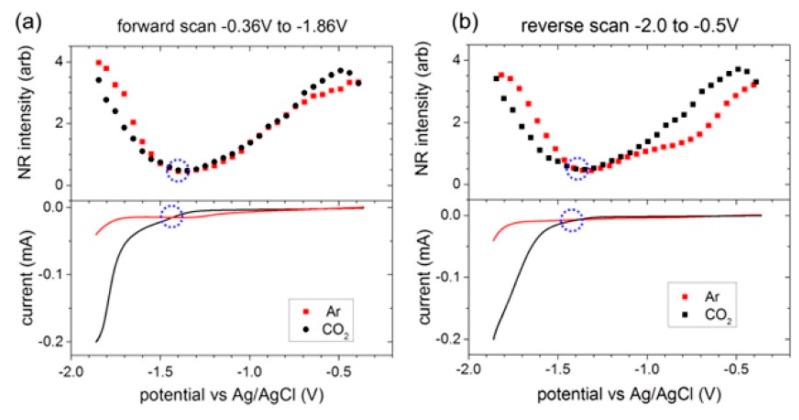
(**Top**) SFG nonresonant (NR) signals when room-temperature ionic liquids (RTILs) electrolytes were saturated with Ar or CO_2_. (**Bottom**) Meniscus cyclic voltammograms (CVs). The circles denote the threshold potential for CO_2_ reduction and the NR intensity minimum. There was a close association between the onset of CO_2_ reduction and the nonresonant intensity minimum. (**a**) First forward (reduction) scan; (**b**) First reverse (oxidation) scan. Reprinted with permission from ref. [54]. Copyright 2015 American Chemical Society.

To explore the potential-dependent structural changes in the interfacial double layer region during the electrochemical reduction of CO_2_, Rey et al. employed a non-resonant (NR) SFG method to study the [Emim][BF_4_] system with 0.3 mol % water on an Ag electrode surface [54]. NR-SFG is similar to NR second-harmonic generation (SHG) measurement which is frequently used in the study of electrified interface system. The authors observed that the NR intensity minima appeared at a similar potential for both the Ar and CO_2_ saturated cases, thereby indicating that the NR intensity changes are closely related to the intrinsic properties of the RTIL itself. As shown in Figure 3, the curvature of the potential-dependent NR intensity was different from the threshold potential for CO_2_ reduction, −1.33V vs. Ag/AgCl in both forward and reverse scans. Therefore, they concluded that the intrinsic structural transition in the RTIL system controls the potential of CO_2_ reduction, and this NR intensity behavior could be a descriptor for the design of new RTIL-based electrochemical systems.

**Figure 4:**
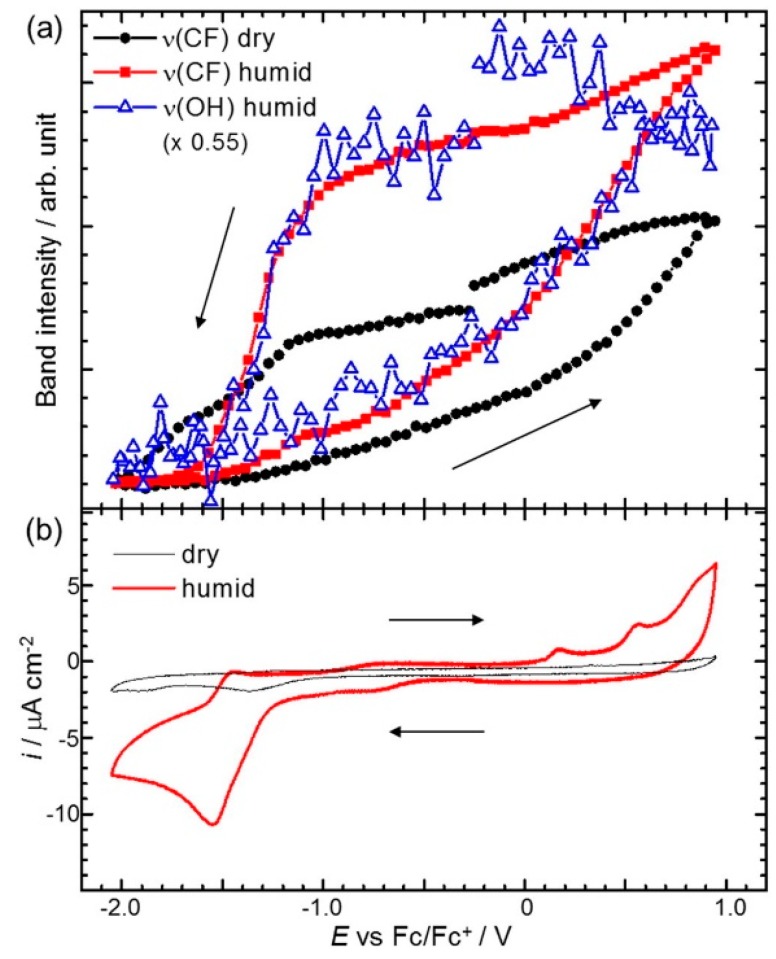
(**a**) SEIRAS band intensities as a function of potential recorded at every 0.05 V interval during a potential scan at 2 mV/s in dry (c_w_ < 1 ppm) and humid (c_w_ = 700 ppm) [Bmim][TFSA]. Band intensities are obtained by direct integration of the bands with respect to a local baseline; (**b**) CVs simultaneously measured with SEIRAS in dry and humid [Bmim][TFSA]. Reprinted with permission from ref. [56]. Copyright 2016 Elsevier. c_w_: water concentration.

Osawa’s group has reported various studies using surface-enhanced infrared absorption spectroscopy (SEIRAS) for probing the RTIL/electrode interface characters. The most representative cases are potential dependent in-situ SEIRAS measurements of the interface between the Au electrode and pure 1-butyl-3-methylimidazolium bis(trifluoromethanesulfonyl)amide ([Bmim][TFSA]) or a [Bmim][TFSA]/water mixture solvent [55,56]. In their measurements, the authors were able to observe the potential-dependent restructuring of the solvent components, such as the cations and anions of [Bmim][TFSA] or the water molecules at the Au electrode surface by direct integration of specific SEIRAS peaks during the cyclic voltammetry measurements. They observed the intrinsic behavior of the [Bmim][TFSA] components at the Au electrode surface in terms of the applied potential and found that the first interfacial layer is dominated by the cations at a negatively charged surface and anions at a positively charged surface. Thus, the mixing and exchanging of ions in the surface layers that occur during the potential scans are accompanied by a certain degree of hysteresis arising from the strong association between ionic components. More interesting behavior has been observed in the [Bmim][TFSA]/water mixture system with a water concentration (c_w_) of 700 ppm. As shown in Figure 4, the band intensity profiles of *v*(OH) from water and *v*(CF_3_) from the [TFSA] plotted against the potential cycle are very similar, which strongly suggests the association of water molecules with [TFSA] at the electrode surface. In addition, the authors suggested that the large intensity changes (hysteresis) in humid conditions compared to the dry state indicate the accelerated cation–anion exchange rate induced by water.

**Figure 5:**
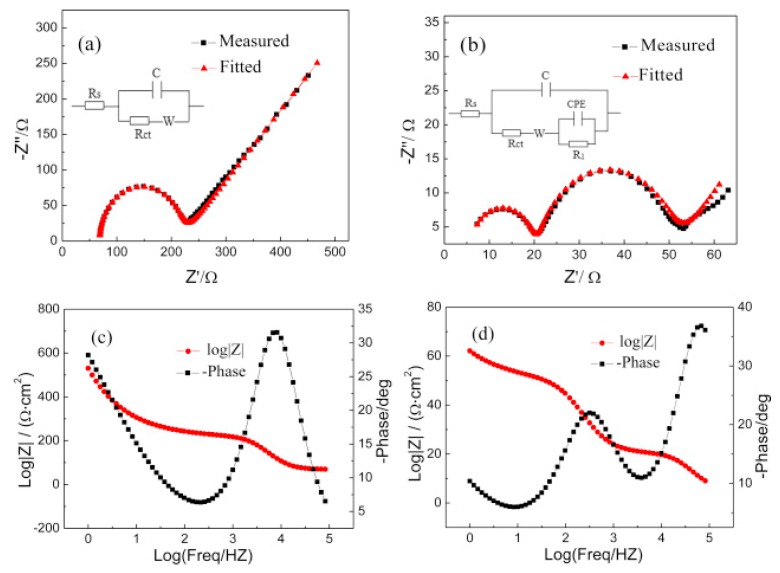
Impedance spectra detected in (**a**) [Bu_4_N][CF_3_SO_3_]/PC and (**b**) [Bmim][CF_3_SO_3_]/PC; Bode plot detected in (**c**) [Bu_4_N][CF_3_SO_3_]/PC and (**d**) [Bmim][CF_3_SO_3_]/PC. Reprinted with permission from ref. [59]. Copyright 2016 Elsevier.

Another useful technique to identify the characteristics of solid–liquid interfaces, especially the quantitative resistance factors in solid–liquid interface system, is electrochemical impedance spectroscopy (EIS) [57,58]. Yang et al. reported that the EIS profile detected in 1-butyl-3-methylimidazolium trifluoromethanesulfonate/propylene carbonate ([Bmim][CF_3_SO_3_]/PC) is clearly distinct from that detected in a tetrabutylammonium trifluoromethanesulfonate/propylene carbonate ([Bu_4_N][CF_3_SO_3_]/PC) solution on an Au electrode, and the differences in EIS analysis explained reasonably well the enhanced performance in [Bmim][CF_3_SO_3_]/PC solution (239 mV positive shift in onset potential) [59]. From the Nyquist plots (Figure 5a,b) and the corresponding fitted equivalent circuit parameters, the authors suggested that the RTIL components are strongly absorbed on the electrode surface, forming a film layer; consequently, RTIL solutions show lower solution and charge transfer resistances. Thus, they concluded that the activation energy and overpotential of CO_2_ reduction are reduced by the interaction of CO_2_ and the RTIL layer formed at the electrode surface.

As summarized above, researchers have carried out mechanistic investigations by experimentally observing the structural changes of the double layer formed at the electrode–liquid interface, and they tried to relate the experimental observations with the electrochemical CO_2_ reduction performance. They commonly observed strong adsorption of cations of RTILs onto the catalyst surface during electrochemical reaction even in low concentration of RTILs, and the detailed structural behaviors of RTIL components showed strong dependency on applied electric potential. It is obvious that the performance of electrochemical reaction is altered by RTIL components in the vicinity of the electrode surface. However, these spectroscopic observations alone may not be enough to elucidate the detailed mechanism due to the complicatedly entangled structural and electrostatic properties of double layer region, which are still barely known.

Beyond such experimental efforts, theoretical studies have also been extensively carried out to elucidate the underlying mechanism of electrochemical CO_2_ reduction in RTIL-based solvents [60,61,62,63,64,65,66,67,68]. In particular, Norskov’s group has focused on the effect of the electric field generated by cations in the vicinity of the electrode surface. Urushihara et al. examined first-principles-based thermodynamic stability (Pourbaix diagram) of [Emim] at the Ag(111) water interface as a function of electrochemical potential and [Emim] concentration. In their simulations, the solvation effect was described using the combination of explicit water molecules, which were modeled adsorbed on the electrode surface, and implicit water, modeled as a dielectric medium [66].

**Figure 6:**
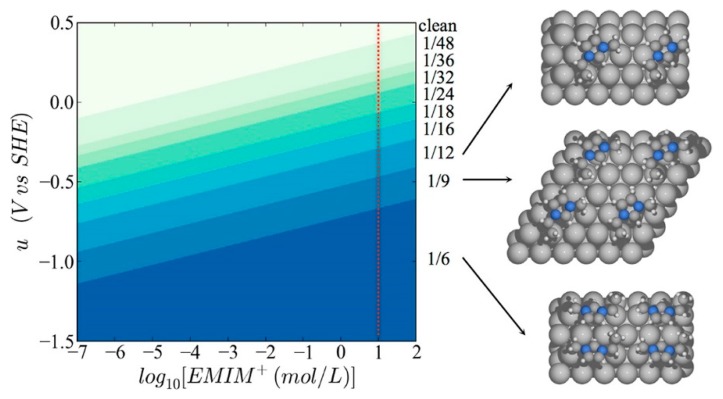
Surface Pourbaix diagram of [Emim] on the silver (111) surface in water. The red dotted line shows experimental conditions of Rosen et al. [33]. The right structures are images of the most stable configurations of adsorbed [Emim]. Reprinted with permission from ref. [66]. Copyright 2015 American Chemical Society.

As shown in Figure 6, they found that the coverage of [Emim] on the Ag(111) surface increased as a more negative bias potential was applied and as the concentration of RTIL in aqueous solution increases. Based on the theoretical Pourbaix diagram, they found that the coverage of [Emim] may be around one-ninth in the experimental condition of Rosen et al.’s work [33]. Thus, they suggested that the strong electric field on the electrode surface could have a positive effect by stabilizing the highly polarizable reaction intermediates, such as CO_2_^−^ or −COOH.

**Figure 7:**
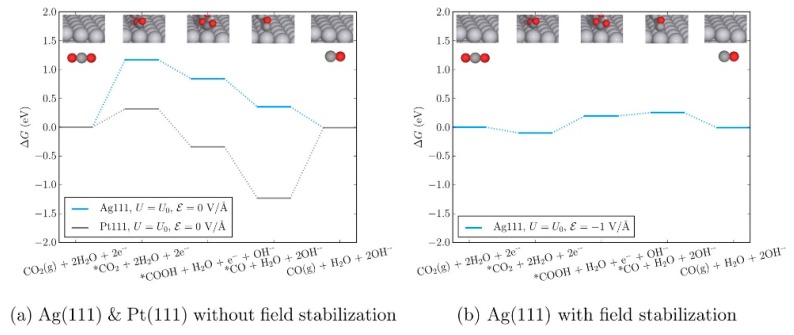
Free-energy diagram for CO_2_(g) to CO(g) without (**a**) and with (**b**) the effects of electric field on Ag(111) at the equilibrium potential of the overall reaction (−0.11 V vs. SHE). The same pathway on Pt(111) is shown to contrast CO_2_ and CO adsorption energies for the two metals in the absence of an applied electric field. Reprinted with permission from ref. [60]. Copyright 2016 American Chemical Society.

Moreover, Chen et al. evaluated the theoretical reaction energetics of CO_2_-to-CO on Ag(111) and Pt(111) electrodes in the presence of the electric field induced by the adsorbed cations [60]. The authors confirmed that the changes in the adsorption energies of the reaction intermediates that arise from the presence of explicit solvent and cations (K^+^ or [Emim]) and can be effectively described by applying an external homogeneous electric field to the simulation cell. Therefore, they developed a micro-kinetic model that includes an external electric field to describe the reaction thermodynamics in the RTIL solvent system. Based on the reaction thermodynamics including the effect of the external electric field, the authors found that the CO_2_ adsorption step and the first reduction step forming *COOH (* denotes the species adsorbed on the catalyst surface) become easier, which explains the experimental observations of Rosen et al. (Figure 7) [33].

Despite some minor discrepancies, there is consensus in previous experimental and theoretical studies regarding the character of the interfacial region and its effect on the reaction energetics: the cations at the electrode surface are the main driving force increasing the activity of RTIL-based electrocatalytic systems. This is also consistent with the recent experimental studies reporting non-RTIL-based cationic field-induced activity enhancements in CO_2_ electrochemical reduction [65,69]. However, a more complicated mechanism for determining the overall activity in RTIL-based systems is required. For example, no direct correlation is found among the chemical details of RTIL components and activity enhancement, indicating that the complex interactions between the RTIL components and the reaction intermediate species must be addressed. In other words, it is necessary to understand the complex physico-chemical nature of RTIL-based electrochemical systems, and thereby we can develop a general design principle for more enhanced RTIL-based electrochemical systems. The simulation method used in the previous theoretical studies is the first-principle density functional theory (DFT) which is known to predict thermodynamic properties of system with a relatively reliable accuracy level, in principle. However, considering the large size of the molecule and huge dynamical degrees of freedom of the RTIL-based solvent system, the computational burden should be concerned when the DFT method is applied. We conceive that the development/application of multiscale simulation methods (e.g., combined quantum mechanical/molecular mechanical (QM/MM) simulation) that can accurately describe the reaction energetics as well as the dynamical and structural characteristics of a complex catalytic system (with affordable computation burden) would be of importance in computational chemistry research field.

## 3. The Roles of RTILs in Chemical Interactions

**Figure 8:**
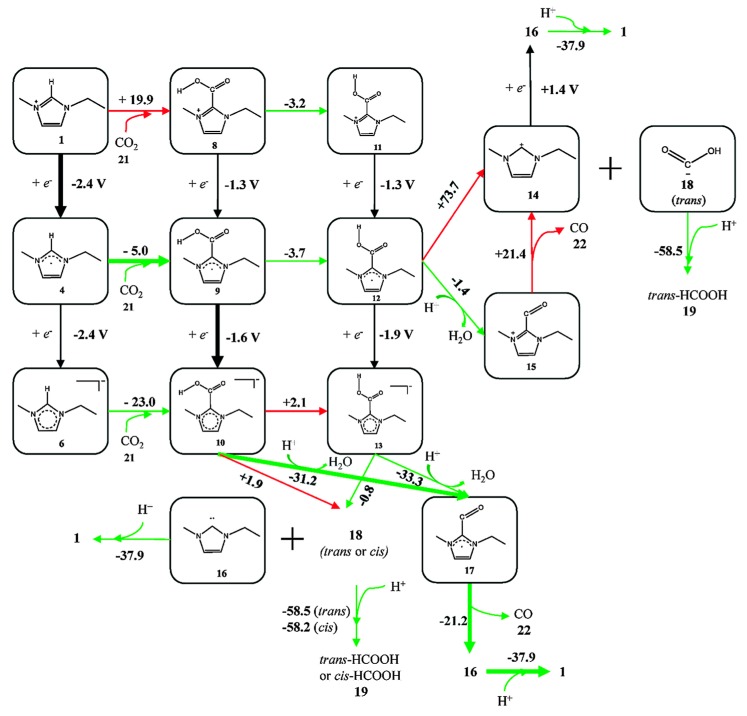
A schematic flow of reaction intermediates. [BF_4_] anions were not shown in the figure for simplicity. The values represent the calculated reaction free energies (kcal·mol^−1^) in each reaction step or standard electrode potential (V vs. SHE) in each electrochemical step. Green arrows represent the thermodynamically favorable pathway and red arrows represent unfavorable ones. Reprinted with permission from ref. [67]. Copyright 2015 Royal Society of Chemistry.

Because experiments are based on ensemble measurements, the collective behavior of the reaction medium is often discussed, as summarized above. However, rather contradictorily, researchers have also tried to find mechanistic clues concerning the activity enhancement in the local intermolecular interactions between reaction intermediates and RTIL components (usually through the acidic hydrogen at the C2 position in imidazolium-based cations [70,71]). Thus, many experimental and theoretical studies have been performed to verify the effect of direct chemical interactions between the reaction intermediates and the RTIL components and to identify the correlation between reactivity and the chemical composition of the RTIL. Using DFT calculations, Wang et al. suggested the most likely reaction pathway from CO_2_ to CO, catalyzed by direct chemical interaction with [Emim][BF_4_] [67]. They found the most probable and stable geometries of a 3-body system comprising [Emim][BF_4_]-CO_2_, and they evaluated the energies of every possible reaction intermediate, resulting in a mechanism for the imidazolium-based catalytic cycle (Figure 8). They argued that there exists a stable interaction between CO_2_ and the RTIL components at the molecular level. However, they found that there was a large discrepancy between the estimated onset potential obtained via DFT calculations, and this was due to the absence of electrode surface effects.

**Figure 9:**
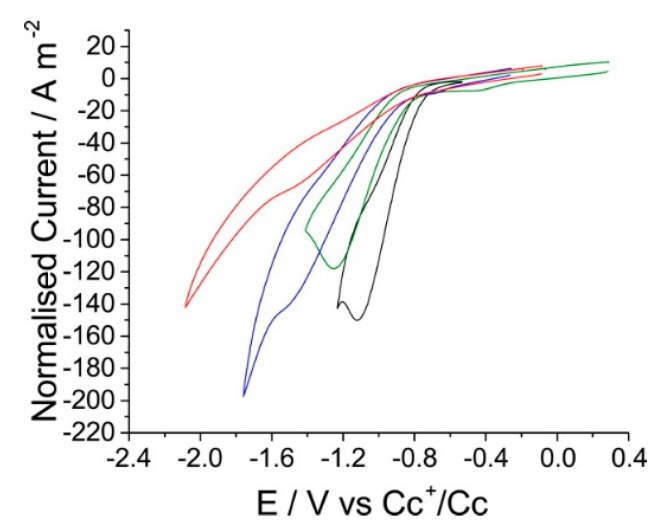
CVs of the reduction of carbon dioxide on silver with a scan rate of 1 V·s^−1^, reported vs. a Cc^+^/Cc reference, in a range of RTILs where the cation is varied. Black is [Bmim][NTf_2_], green [Bmpyrr][NTf_2_], blue [Emim][NTf_2_], and red [Pmim][NTf_2_]. Reprinted with permission from ref. [72]. Copyright 2016 American Chemical Society.

In contrast, many experiments have been carried out to compare the CO_2_ reduction activities of various combination of cations and anions of RTILs to decipher the underlying mechanism and to screen and optimize the various combinations. Tanner et al. screened combinations of metal electrodes (Ag, Au, Pt, and glassy carbon), RTIL cations (1-butyl-1-methylpyrrolidinium ([Bmpyrr]), [Emim], 1-propyl-3-methylimidazolium ([Pmim]), and [Bmim]), and anions (bis(trifluoromethylsulfonyl)imide ([NTf_2_]), tris(pentafluoroethyl)trifluorophosphate ([FAP]), and [BF_4_]) [72]. They confirmed that Ag was the most active electrode material in [Bmim][NTf_2_], and they then screened combinations of RTIL cations and anions mentioned above with an Ag electrode. They observed distinct, cation-dependent changes in the CO_2_ reduction activity (when combined with the [NTf_2_] anion), showing the following trend: [Bmim] > [Bmpyrr] > [Emim] > [Pmim] (Figure 9). The chain length of the imidazolium cation had a less systematic effect, and the pyrrolidinium cation, which has no acidic protons, showed higher activity than the imidazolium cations [Emim] or [Pmim]. In addition, the performance change on varying the anion showed the following trend: [NTf_2_] > [BF_4_] > [FAP], which could not be simply explained by solubility (CO_2_ solubility order: [FAP] > [NTf_2_] > [BF_4_] [73,74]). Thus, the authors suggested that the RTIL components play a complex role in the CO_2_ reduction mechanism, presumably altering the character of the double-layer at the electrode surface, which contrasts with the prevailing simplified view focusing on the local chemical interaction between CO_2_ and RTIL components or CO_2_ solubility.

Zhao et al. reported a more extensive screening study of 13 ionic liquids or salts, including imidazolium, pyrrolidinium, ammonium, phosphonium, borate, pyridinium, and sulfonium, and 7 ammonium salts with an Ag electrode [75]. They performed electrochemical measurements in the presence of a low concentration of the above compounds (2.0 mM) dissolved in an aprotic (acetonitrile) solution of 0.1 M tetrabutylammonium hexafluorophosphate ([Bu_4_N][PF_6_]) to minimize background effects from pure solvents. They observed that most compounds enhanced the catalytic CO_2_ reduction kinetics, but imidazolium and pyrrolidinium cations were the most active. However, pyrrolidinium-based RTILs showed better performance than those containing imidazolium in terms of long-term stability, which is ascribed to the different role of cations; pyrrolidinium is believed to change the character of the interfacial double layer, while imidazolium is believed to act as a co-catalyst.

**Figure 10:**
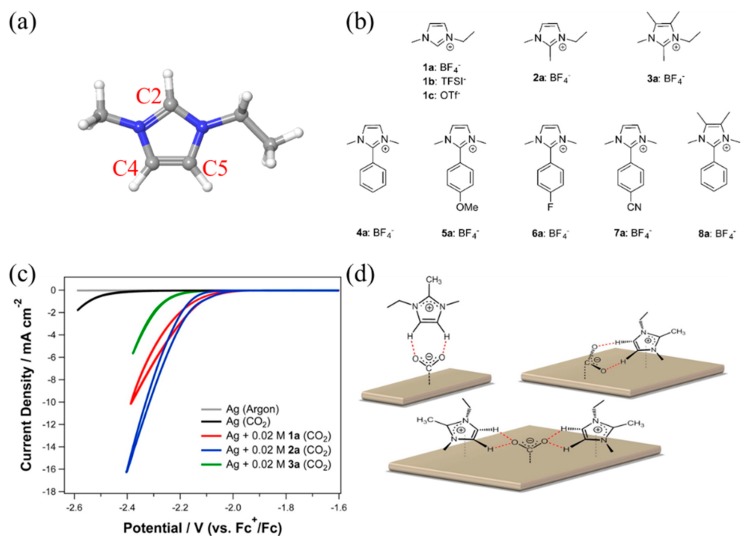
(**a**) Molecular structure of imidazolium cation; grey is carbon, white is hydrogen and blue is nitrogen; (**b**) Chemical structures of various functionalized imidazolium salts studied; (**c**) Effect of different cations (1a, 2a, 3a) on the electrochemical reduction of CO_2_; (**d**) Possible binding modes of 2a with an electro-generated CO_2_ anion radical on a silver surface. Hydrogen bonds represented by dashed line (red). Reproduced with permission from ref. [76]. Copyright 2016 American Chemical Society.

Lau et al. performed another interesting screening experiment, and they concluded that the activity enhancement of imidazolium-based RTILs originates from the C4 and C5 hydrogen atoms rather than that at the C2 position (Figure 10a) [76]. They systematically synthesized various functionalized imidazolium-based RTILs, as shown in Figure 10b, and observed that the methylation of the C2 hydrogen did not affect the catalytic activity, while the methylation of C4 and C5 hydrogen significantly decreased the activity (Figure 10b,c). Thus, they suggested that the coordination of the reaction intermediates and the C4 and C5 hydrogen results in the stabilization of the intermediates (Figure 10d). They also screened various imidazolium-based RTILs functionalized at the C2 hydrogen and found that RTIL 4a in Figure 10b showed the best activity and stability. Interestingly, they showed a dramatic loss of activity after methylation of the C4 and C5 positions of RTIL 4a (8a in Figure 10b), which is consistent with their conclusion; that is, the formation of interactions via the C4 and C5 hydrogen atoms stabilizes the intermediates, enhancing the activity.

**Figure 11:**
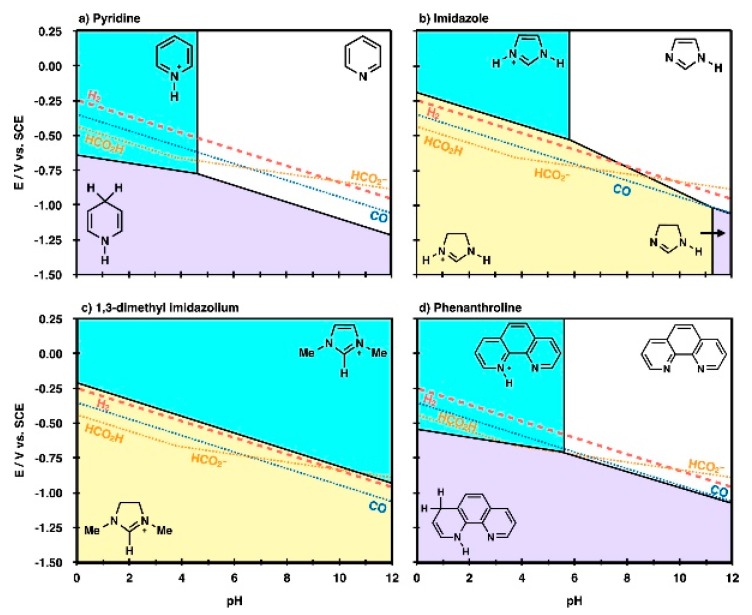
Molecular Pourbaix diagrams for (**a**) pyridinium, (**b**) imidazolium, (**c**) 1-ethyl-3-methyl imidazolium and (**d**) phenanthroline species. The diagrams depict electrochemical conditions (variable pH and *E*), where nonprotonated (white region), monoprotonated (cyan region), 2H^+^ + 2e^−^ reduced (purple region), and 3H^+^ + 2e^−^ reduced (yellow region) are the thermodynamically most stable states in water solvent. Overlaid in the diagrams are standard reduction potentials (SRPs) for 2H^+^ + 2e^−^ → H_2_ (H_2_, red dashed line), CO_2_ + 2H^+^ + 2e^−^ → CO + H_2_O (CO, blue dotted line), CO_2_ + 2H^+^ + 2e^−^ → HCO_2_H (HCO_2_H) and CO_2_ + H^+^ + 2e^−^ → HCO_2_^−^ (HCO_2_^−^, gold dotted lines). At applied potentials, more negative than these lines, product species would be thermodynamically stable compared with reactant protons, electrons, and CO_2_. Reprinted with permission from ref. [62]. Copyright 2015 American Chemical Society.

From the above experimental screening studies, it can be found that the performance of RTIL based CO_2_ reduction system is determined by not only the individual role of each component but also the complex reaction environment around the active site. Beyond the local interactions between the reaction intermediate species and the RTIL components, many studies have focused on the thermodynamic properties of the RTILs themselves to provide an overall understanding of RTIL-containing reaction systems [62,63,68]. Marjolin et al. reported that it is possible to estimate the reactivity of electrochemical CO_2_ reduction with aromatic *N*-heterocycles (ANH) by evaluating the Pourbaix diagram of ANHs in terms of the pH and electrode potential [62]. The authors showed that the triple point of a DFT-based molecular Pourbaix diagram successfully reproduced the experimental CO_2_ reduction conditions (Figure 11). Thus, they proposed that the theoretical Pourbaix diagram triple point could be a unifying descriptor to estimate which molecules would be good proton or hydride shuttling agents.

## 4. Conclusions

**Figure 12:**
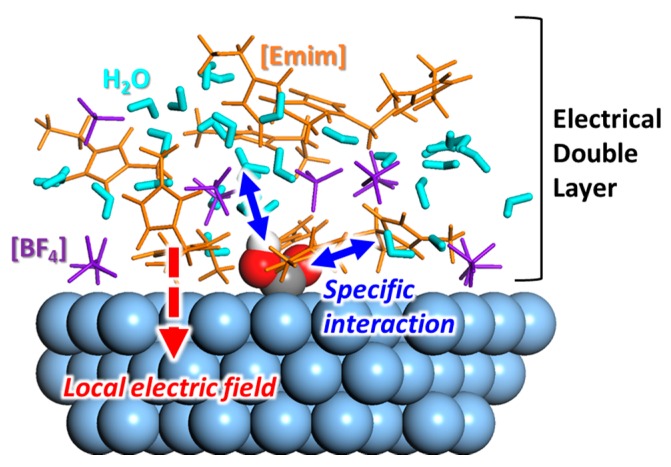
Schematic molecular structure model of *COOH in [Emim][BF_4_]/water mixture solvent. The solvent containing RTIL components forms a complex and unique electrical double layer on catalyst surface, which is conceived to affect electrocatalytic activity.

So far, many experimental studies have reported the improved performance of RTIL-based electrocatalytic systems for CO_2_ conversion. It is believed that the unique physicochemical properties of the RTIL components enhance both the activity and selectivity. To develop a more advanced RTIL-based electrocatalytic system, we must find the underlying mechanisms that can be used to guide component design. Experimental studies have mainly focused on the observation of the structural characteristics (mainly physical) of the RTIL components at the electrode–solvent interface. The cationic species are primarily located at the electrode surface during electrolysis, interacting with the reaction intermediates or altering the character of the electric double layer (summarized in Figure 12). Along with these experimental efforts, theoretical works have mainly focused on quantitative thermodynamic analyses of the physicochemical interactions between the RTIL components and the reaction intermediates, primarily at the electrode–solvent interface or in the bulk region. In addition to deciphering the underlying mechanisms, several screening studies have been conducted on various combinations of RTIL components to screen and optimize the combinations.

Despite various in-depth systematic research efforts, unfortunately, we have found that there is still lack of general consensus about the actual role of RTIL components in electrochemical systems among various experimental reports, among theoretical investigations, and even between experiments and simulations. This limits researchers in deriving the key major factors determining the catalytic activity of the electrocatalytic system, the so-called descriptors. We speculate that this is due to the complicated entangled structural and electrostatic properties of RTIL at the double layer region. Therefore, we conclude that future studies will need to put research efforts on identifying not only the role of each ionic component of an RTIL separately, but also the cooperative and collective role of media in a physical and chemical manner at the electrode surface or in the region above the surface. We anticipate arriving at the RTIL design principle based on such fundamental efforts to characterize the physical, chemical, and thermodynamic properties of complex RTIL-based electrocatalytic systems in near future.

## Abbreviations

The following abbreviations are used in this manuscript.

[Emim]	1-Ethyl-3-methylimidazolium
[Bmim]	1-Butyl-3-methylimidazolium
[Pmim]	1-Propyl-3-methylimidazolium
[Bmpyrr]	1-Butyl-1-methylpyrrolidinium
[Bu_4_N]	Tetrabutylammonium
[BF_4_]	Tetrafluoroborate
[PF_6_]	Hexafluorophosphate
[MS]	Methylsulfate
[TFSA]	Bis(trifluoromethanesulfonyl)amide
[CF_3_SO_3_]	Trifluoromethanesulfonate
[NTf_2_]	Bis(trifluoromethylsulfonyl)imide
[FAP]	Tris(pentafluoroethyl)trifluorophosphate
